# Reduced Multidrug Susceptibility Profile Is a Common Feature of Opportunistic *Fusarium* Species: *Fusarium* Multi-Drug Resistant Pattern

**DOI:** 10.3390/jof3020018

**Published:** 2017-04-10

**Authors:** Saad J. Taj-Aldeen

**Affiliations:** 1 Mycology Unit, Microbiology Division, Department of Laboratory Medicine and Pathology, Hamad Medical Corporation, P.O. Box 3050 Doha, Qatar; saadtaj51@gmail.com; Tel.: +974-55050342; 2 Weill Cornell Medicine-Qatar, P.O. Box 3050 Doha, Qatar

**Keywords:** *Fusarium*, fungemia, disseminated infections, local infections, underlying conditions, antifungal susceptibility, multi-drug resistance, Amphotericin B, voriconazole

## Abstract

The resistance among various opportunistic *Fusarium* species to different antifungal agents has emerged as a cause of public health problems worldwide. Considering the significance of multi-drug resistant (MDR), this paper emphasizes the problems associated with MDR and the need to understand its clinical significance to combat microbial infections. The search platform PubMed/MEDLINE and a review of 32 cases revealed a common multidrug-resistant profile exists, and clinically relevant members of *Fusarium* are intrinsically resistant to most currently used antifungals. Dissemination occurs in patients with prolonged neutropenia, immune deficiency, and especially hematological malignancies. Amphotericin B displayed the lowest minimum inhibitory concentrarions (MICs) followed by voriconazole, and posaconazole. Itraconazole and fluconazole showed high MIC values, displaying in vitro resistance. Echinocandins showed the highest MIC values. Seven out of ten (70%) patients with neutropenia died, including those with fungemia that progressed to skin lesions. Clinical *Fusarium* isolates displayed a common MDR profile and high MIC values for the most available antifungal agents with species- and strain-specific differences in antifungal susceptibility. Species identification of *Fusarium* infections is important. While the use of natamycin resulted in a favorable outcome in keratitis, AmB and VRC are the most used agents for the treatment of fusariosis in clinical settings.

## 1. Introduction

*Fusariuma* is a serious opportunistic human pathogen affecting immunocompromised patients [1,2] and represents the second most common cause of filamentous fungi infections after aspergillosis [3,4]. *Fusarium* species cause a broad spectrum of infections in humans; the invasive and disseminated infections occur predominantly in severely immunocompromised individuals [1,5]. These infections may manifest as a fever that does not respond to antimicrobial therapy [6]. Underlying diseases for development of invasive fusariosis are hematologic malignancies, hematopoietic cell transplantation, neutropenia, and impaired cellular immunity [5,6,7,8,9]. Infections may also occur in immunocompetent individuals [10]. Localized infections, such as onychomycosis, keratitis or endopthalmitis and other skin infections, are frequently manifested in immunocompetent individuals and are often associated with previous trauma [11,12,13,14,15]. *Fusarium* species are genetically diverse and are commonly environmental organisms including phytopathogens, saprophytes and those isolated from water systems [1,16,17]. Although *Fusarium* species are plant pathogens, they may occasionally cause infections in animals [18,19], *Fusarium* from veterinary sources was previously known to infect humans [20]. This hyaline hyphomycete fungus poses a challenge for human disease management because conidia can disperse in the atmosphere over a long distance and infect a new susceptible host [21].

*Fusarium* species are among the most resistant fungi; infections are commonly refractory against treatment with the most known systemic and conventional antifungal agents [22]. Estimated mortality rates of 50–75% in disseminated infections may arise, especially in immunosuppressed patients [1,5,8,23]. *Fusarium* pathogens typically show broad in vitro resistance to antifungal agents with a high variability being present within each species [24,25,26,27,28,29]. In vitro susceptibility testing may represent a tool for the selection of an appropriate therapy. In general, members of the *Fusarium solani* species complex (FSSC) are most commonly observed in all clinical infections and show the highest minimum inhibitory concentrations (MICs) against various antifungal drugs. Amphotericin B (AmB) is the most active drug followed by voriconazole (VRC) [27,29,30]. Members of the *Fusarium fujikuroi* species complex (FFSC), which are responsible for approximately one-third of the disseminated infections, display a similar susceptibility profile [25]. Based on such in vitro susceptibility data for various *Fusarium* species, the optimal treatment options remain limited and controversial. ESCMID and ECMM joint guidelines suggested that early therapy with VRC or lipid-based (LAmB) in conjunction with surgical intervention is of utmost importance to prevent dissemination [31]. Most of the available MIC data on AmB and triazoles have been reported for a variety of *Fusarium* species, using the CLSI-based methods [24,28,32,33]. This paper reviews the most recent cases and discusses the susceptibility and multidrug resistant patterns of clinically important *Fusarium* species in response to the most available systemic antifungal agents.

## 2. Definitions

**Figure 1:**
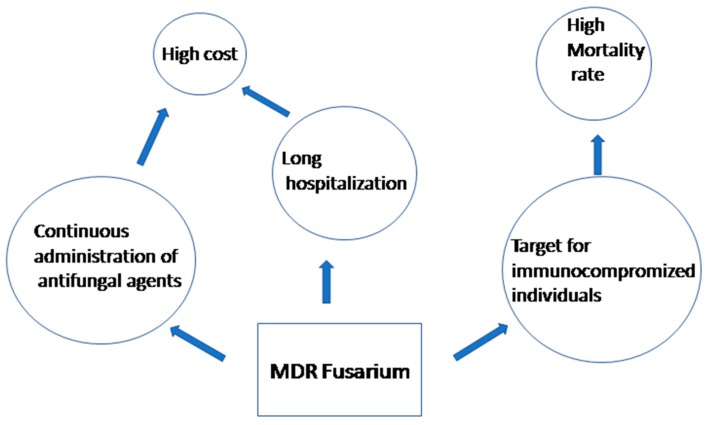
Clinical and economic factors associated with MDR *Fusarium* infections.

Invasive *Fusarium* infection (fusariosis) is infection with at least one positive blood culture or the isolation of the same strain from two or more body sites [29]. Localized diseases, as defined previously [29], are infections of the skin, nail, cornea and wound without deep tissue involvement. Proven *Fusarium* infection requires the visualization of fungal elements by direct microscopy of the clinical specimens, the isolation of the fungal etiology in culture, and compatibility with infectious disease processes [34]. Multidrug resistance (MDR) can be defined as the broad-spectrum tolerance to antifungal agents; more precisely, the phenotype of non-susceptibility to at least one agent from two antifungal classes (e.g., azoles and echinocandins). Antifungal resistance is associated with a high mortality rate and increases the length of hospital stays, resulting in a high cost (Figure 1). MDR resistance decreases the efficacy of treatment, and hence, results in a prolonged time of infection. MDR resistance in *Fusarium* can be classified into:

(i) Intrinsic resistance: the innate ability of a *Fusarium* species to resist activity of an antifungal agent through its inherent structural or functional characteristics without prior exposure to the drug, which allows tolerance of a drug or antifungal class. It occurs naturally in *Fusarium* species that have never been susceptible to that agent [25,35,36].
(ii) Acquired resistance: used to describe the resistance that arises in *Fusarium* after exposure to the antifungal agent. The development of resistance and infection recurrence after drug discontinuation [37] and fungal breakthrough infections associated with posaconazole prophylaxis [38] suggest the emergence of resistant strains.
(iii) Clinical resistance: a situation in which the infecting *Fusarium* species is inhibited by the concentration of an antifungal agent that is associated with therapeutic failure or reappearance of infections. Such failure can be attributed to a combination of factors related to impaired host immune function, *Fusarium* species, or the antifungal agent [39,40].

## 3. Clinical Significance

**Figure 2:**
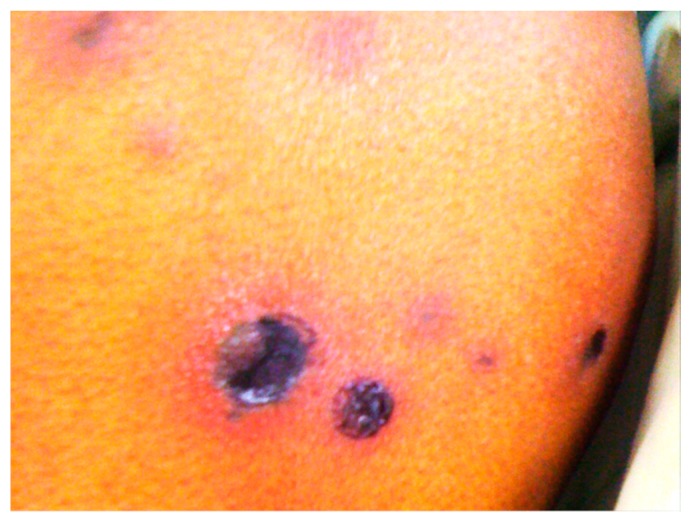
Erythematous cutaneous lesions on the thigh of a 24-year-old T-cell lymphoma female with neutropenia (<100 cells/µL) who died from *F. solani* fungemia irrespective of antifungal treatment (Case #15 Table 1 and Table 2). Photography by Saad J. Taj-Aldeen (Hamad Medical Corporation, Qatar).

*Fusarium* species cause a broad spectrum of infections in humans. The focus will be on two devastating infections caused by *Fusarium* species: disseminated fusariosis, which may lead to an unfavorable outcome, and keratitis, which causes injury that may progress to the loss of sight. *Fusarium* has been found in environmental samples and is widely distributed in soil, plants, plant debris and other organic substrates, as well as in water systems, including the plumbing systems of hospitals [41]. However, it was demonstrated that the infection is a community rather than hospital acquisition, based on a study that investigated the environmental sources of *Fusarium* infections in a tertiary-care center [42]. The first case of disseminated fusariosis was described in 1973 [43]. Since then, there was a significant increase in the occurrence of disseminated disease, probably reflecting the increase in the number of immunocompromised hosts. Reports have focused on two primary groups: those with hematologic malignancies, susceptible secondarily to neutropenia, and bone marrow transplant recipients. Disseminated infection is characterized by persistent fever not responding to antibiotic treatment and by diffuse metastatic skin lesions with a dark purple central necrosis surrounded by an erythematous ring (Figure 2). The cutaneous lesions are observed in approximately 85% of patients with disseminated *Fusarium* infections and often occur at an early stage of the disease [44], which is the most frequent presentation of disseminated fusariosis [1,3,5,36]. Patients with disseminated fusariosis have an unfavorable prognosis, and the survival rate may reach 33% [45]. Dissemination may proceed to cause intracranial infection; many proven *Fusarium* brain abscess infections mostly in patients with cancer or hematological disorders were reported in the literature [10,46,47,48,49]. *Fusarium* may cause serious osteomyelitis infections in immunosuppressed patients [50]. A review of bone and joint infections by filamentous fungi revealed (*n* = 14; 9.6%) cases caused by *Fusarium* species [51].

Although not completely elucidated, the role of the innate immunity and particularly the Toll-like receptors and T-cell defenses seems to be crucial in the progression of fusariosis [1]. The portal of entry of *Fusarium* species is often not clear. Possibilities include the respiratory tract, gastrointestinal tract, or catheter tip [52]. A toenail infection, or paronychia, in some predisposed individuals, may be the source of disseminated infection [53,54,55,56]. The presence of infections involving the skin or nails should be carefully investigated before initiating immunosuppressive therapy since it has been shown that such lesions can be a focal point for *Fusarium* dissemination.

Keratitis is still the most common infection caused by *Fusarium* species; the incidence is increasing in many areas of the world, especially in tropical areas [57,58,59] and in the USA [12,60]. Farm workers at are greater risk of corneal injury and exposure to airborne conidia [61]. Another risk factor for *Fusarium* keratitis is contact lens wearers and contact lens care solution, as in the Multistate outbreak of *Fusarium* keratitis, which was investigated by the CDC in 2006 [12]. The emergence of novel opportunists within the genus *Fusarium* has frequently been reported [62].

## 4. Susceptibility to Amphotericin B and Voriconazole and Clinical Response

Finding the optimal treatment strategy is a challenge because *Fusarium* species show reduced susceptibility to the most available antifungal agents. The reverse of immunosuppression [41] by the administration of granulocyte-macrophage colony stimulating factor (GM-CF), which is sufficient to render blood culture negative with relief of neutropenia [29], and simultaneous treatment with VRC or L-AmB, is recommended [31]. An indication of the potential correlation between MICs for *Fusarium* species and response to treatment was only found in a recent report investigating CLSI-based MICs for seven *Fusarium* isolates, and the clinical response was documented for patients with invasive fusariosis [42]. *Fusarium* susceptibility to VRC is variable [25,29,43]; several out of 16 *F. proliferatum* strains investigated displayed high MICs against VRC (*n* = 6) with values >16 µL/mL [30], and breakthrough infection has been reported in 16 out of 44 patients with invasive fusariosis—69% were receiving prophylaxis with VRC (8/16; 50%) or posaconazole (POS) (3/16; 19%) [23].

AmB was most active agent against clinical and reference strains in vitro with MICs ranging from 0.062–2 µg/mL [29], whereas an MIC range from 1 to 8 µg/mL was reported for most species and may not be related to clinical outcomes [25,36]. This suggests that the role of any in vitro data for AmB is controversial. Treatment failed in one of the two patients with disseminated fusariosis who received AmB therapy [29]. Important parameters may influence the outcome of an infection, e.g., drug doses, treatment duration, and drug serum levels.

Due to the poor prognosis obtained with monotherapy, combination therapy may be considered in severe *Fusarium* species infections. In vitro susceptibility of AmB plus VRC has shown favorable results [44], in addition to immunocompromised patients [21,45]. In vitro combination of antifungal activity, of natamycin and VRC for fungal keratitis displayed 70% synergistic effects against a significant number of isolates [46]. Combination therapy for disseminated fusariosis in immunocompromised patients was previously reviewed [47]; the patients in 14/20 (70%) cases had favorable treatment responses. All patients in these 14 case reports had underlying hematologic diseases, among which four (29%) underwent hematopoietic stem cell transplantation prior to the disseminated infection. The most common combination regimens used in these 14 cases with successful responses were L-AmB plus VRC (5/14, 36%), followed by AmB deoxycholate plus VRC (2/14, 15%) and L-AmB plus terbinafine (2/14, 15%) [47].

**Figure 3:**
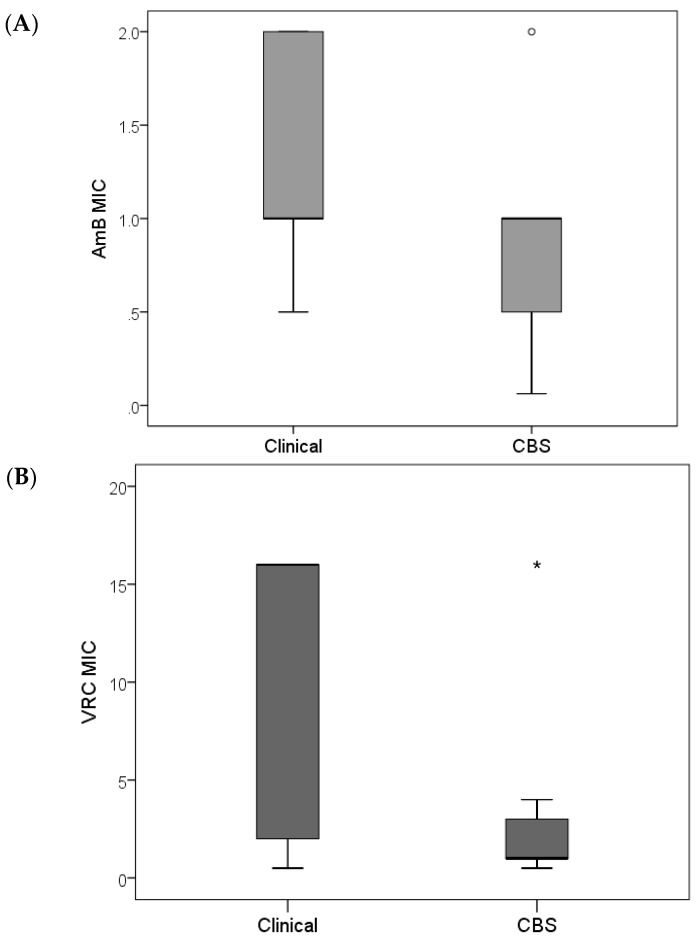
Mann-Whitney *U* and Kruskal-Wallis tests. Box plot distribution of MIC values of amphotericin B (**A**) and voriconazole (**B**) for clinical (39 isolates) and CBS (Westerdijk Institute) reference *Fusarium* strains (12 isolates), showing significantly (*p*-value < 0.05) lower MIC values of the CBS reference strains. Outliers, values that do not fall in the inner fences; (°) cases with values between 1.5 and 3 times the interquartile (IQ) range, i.e., beyond the whiskers; (*) values more than three times the height of the boxes (extremes are cases with values more than 3 times the IQ range).

In vitro antifungal susceptibility of clinical *Fusarium* species revealed that AmB displayed a lower MIC compared with VRC, and reference *Fusarium* species exhibited lower MIC values than the clinical isolates (Figure 3). As concluded earlier [29], this is probably due to previous exposure to antifungal therapy in clinical settings, as most of the Westerdijk Fungal Biodiversity Center (previously CBS) reference strains were isolated from the environment and collected in the era of pre-antifungal use. Interestingly, the two strains from the Westerdijk Fungal Biodiversity Center of human origin displayed high MIC values to the antifungal agents tested [29].

The outcome is improved in disseminated fusariosis; there has been a 21% increase in the survival rate in the last decade due to changes in the treatment practice by shifting therapy to L-AmB and VRC [48].

## 5. Multi-Drug Resistant Cases

The prognosis may be not always favorable when *Fusarium* species exhibit a reduced susceptibility profile. To identify infections caused by *Fusarium* species, the PubMed/MEDLINE database was searched using the keywords, *Fusarium* infections AND fungemia, disseminated fusariosis, *Fusarium* susceptibility and *Fusarium* keratitis for the years “Jan. 2011—Feb. 2017”. Reports included cases in the final analysis with data on the site of infection, underlying disease, etiologic agent, antifungal susceptibility and medical and surgical therapy. Exclusion criteria were cases of non-English literature, incomplete identification of the etiologic agent of the disease, cases missing full text, and cases without *Fusarium* susceptibility data. Descriptive statistics were used to summarize all demographic and clinical characteristics of the patients, and *p* values <0.05 were considered as statistically significant. Analysis was performed using the statistical packages SPSS 19.0 (SPSS Inc. Chicago, IL, USA). The study was approved by the local ethics committee, Medical Research Center (MRC) at Hamad Medical Corporation, Project #16149/16.

Thirty-two of the most recent proven cases of invasive and localized fusariosis were reviewed from the literature. Information pertaining to the source of *Fusarium* species isolation, demographic and clinical data of the patients yielding these isolates is provided in Table 1. Twenty (62.5%) of the cases were proven invasive fusariosis, including fungemia and disseminated infections, and 12 (37.5%) were localized *Fusarium* infections, including mycetoma, keratitis, skin, and onychomycosis. Etiologic agents were *F. solani* species complex (FSSC) *n* = 19, *F. fujikuroi* species complex (FFSC) *n* = 12, and *F. oxysporum* species complex (FOSC) *n* = 1. The patients were treated with AmB, VRC or a combination treatment with a variable degree of responses, and the mortality was 60% (*n* = 12/20) of the total invasive disease cases irrespective of antifungal therapy. Underlying conditions for invasive infections were hematological malignancy, transplant patients, and autoimmune diseases, and ten patients were receiving prophylaxis with antifungal drugs at the time of onset of the invasive fusariosis (Table 1). Seven out of ten (70%) patients with neutropenia died, including those with fungemia that progressed to skin lesions. The reported mortality might be 100% for persistent neutropenic patients with disseminated lesions [1] and 50%–75% for patients with disseminated fusariosis [63].

The in vitro susceptibility pattern of *Fusarium* isolates obtained from these cases showed a multidrug resistant profile (Table 2). Although the new echinocandins drugs such as anidulafungin, micafungin and caspofungin are very important for treating common *Aspergillus* and *Candida* infections, they are inactive for *Fusarium* species [25,64,65,66,67], except for *F. temperatum* case # 21 (Table 2). Hence, it is not recommended in the clinical settings to waste efforts testing *Fusarium* species against echinocandins agents. Although we lack clinical break points for *Fusarium* species and antifungal agents, CLSI epidemiological cutoff values (ECVs) were established for members of the more common *Fusarium* species complexes [68]. The lowest MIC of 0.5 µg/mL AmB was recorded for 7 cases (Table 2), and all FSSC isolates showed MICs within the wild type range ≤ECV value (8 µg/mL) for AmB [68]. The two isolates of *F. verticillioides* (cases 22 & 31) showed high MICs for AmB, above the ECV (4 µg/mL), but case 22 exhibited a low MIC for VRC (1 µg/mL) which is within the wild type range, lower than the reported ECV value for this species (4 µg/mL) [68]. All 19 isolates of the reported FSSC fell within the wild type range for VRC (ECV value = 32 µg/mL), whereas, 11/13 isolates of FSSC tested for POS showed MIC values within the wild type range (≤ the ECV value 32 µg/mL). Overall, FLC and ITC showed poor activity with high MIC values for all the reported cases.

The new triazole antifungal, isavuconazole (ISV), was recently granted approval by the US Food and Drug Administration and the European Medicines Agency for the treatment of invasive aspergillosis and mucormycosis. A randomized, double-blind comparison trial for the treatment of invasive aspergillosis found ISV noninferior to voriconazole. Evaluating the efficacy of ISV in the treatment of mucormycosis revealed comparable response rates to AmB and POS [69,70]. ISV MICs were reported for 7 cases (Table 2) with a range of 4– > 16 µg/mL; there is no controlled trial on the use of ISV in invasive fusariosis, but the MIC values reported for these cases are not encouraging.

## 6. Conclusions

Successful treatment of invasive disease was achieved with the use of AmB and its liposomal form, VRC or combination therapy in addition to GM-CF. In some patients, treatment failed, but others improved (Table 1). Although they are the recommended agents in the ESCMID and ECMM joint guidelines [31], the efficacy of AmB and VRC for treating invasive fusariosis is still controversial as the percentage of patients cured in the cases of Table 1 and in different clinical trials is low [22]. It is difficult to draw a clear correlation of in vitro and in vivo obtained data since many factors influence the outcome of an infection, such as, treatment duration, drug doses, and drug serum level, which are all important parameters. Members of FSSC were the main agents of invasive infections, and they are less susceptible to VRC; these observations agree with the data of a previous report [29]. AmB is the most active agent against *Fusarium* species; clinical *Fusarium* isolates displayed a common MDR profile and high MIC values for the most available antifungal agents with species- and strain-specific differences in antifungal susceptibility. Species identification of *Fusarium* infections is important and may be erroneous or missed in many diagnostic laboratories, which can greatly affect the choice of an appropriate antifungal therapy. These observations emphasize the need to further understand the mechanism of *Fusarium* resistance to combat invasive infections.

## Figures and Tables

**Table 1:** Demographic and clinical data obtained by review of the most recent (2011–2016) proven case series of fusariosis with MDR-resistant *Fusarium* species.

Case No.	Age/Gender	Organism	Underlying Disease	Neutropenia	Prophylaxis	Infection	Treatment	Outcome	Ref. No.
1	32/M	*F. ramigenum*	Common variable immunodeficiency	No	No	Invasive lung infection	4 mg/kg IV q12h VRC for 6 months and continuous immunoglobulin substitution with 25 g/d, (5 d/month)	Survival	[71]
2	21/M	*F. petroliphilum*	Aplastic anemia	Yes	POC (200 mg three times/d)	Fungemia/skin lesions	L-AmB lipid complex 4 mg/kg/d, then VRC 200 mg/d + granulocyte transfusion	Died	[72]
3	44/M	*F. solani*	AML	Yes (<500/µL)	POC (600 mg/d)	Fungemia/skin lesions, Lung infections	L-AmB (5 mg/kg/day) + VRC (4 mg/kg/d; 6 mg/kg/first day loading dose).	Died	[9]
4	64/M	*F. keratoplasticum*	None	No	No	Mycetoma, right ankle	ITC 400 mg/day (14 months), then terbinafine 250 mg/d for 10 months	Survival	[73]
5	37/M	*F. pseudensiforme*	None	No	No	Mycetoma, left foot	Oral ITC (400 mg/d)	Improved, lost follow up after six months	
6	17/M	*F. oxysporum*	None	No	No	Ecthyma gangrenosum	VRC (400 mg/d orally + surgical debridement	Improved/lost follow up	[74]
7	46/M	*F. solani*	None	No	No	keratitis	Topical 1% VRC + 5% natamycin, +0.01 mg AmB + systemic VRC		[75]
8	65/M	*F. solani*	T cell large anaplastic lymphoma erythroderma without systemic involvement	No	FLC (200 mg IV BD)	Fungemia	AmB (20 mg IV OD)	Died	[76]
9	60/F	*F. sacchari*	None/trauma with sugar cane leaf	No	No	keratitis	Oral ITC/topical VRC/Keratoplasty	Responded to the treatment	[77]
10	45/M	*F. sacchari*	None/trauma with sugar cane leaf	No	No	keratitis	Oral ITC/topical VRC +AmB/Keratoplasty	Responded to the treatment	
11	40/M	*F. sacchari*	None/trauma with sugar cane leaf	No	No	keratitis	Topical VRC/Keratoplasty	Responded to the treatment	
12	60/F	*F. sacchari*	None/trauma with vegetative matter	No	No	keratitis	Oral ITC/topical VRC + natamycin	Responded to the treatment	
13	80/F	*F. petroliphilum*	Autoimmune disease on corticosteroids	Yes	No	Fungemia	FLC (empiric)	Died	[29]
14	37/M	*F. petroliphilum*	AML	Yes	No	Fungemia	AmB + GM-CSF	Recovered	
15	24/F	*F. solani sensu lato*	T-cell lymphoma	Yes	?	Fungemia/skin lesions	AmB + VRC + GM-CSF	Died	
16	64/M	*F. falciforme*	AIDS	No	No	Toe nail Onychomycosis	ITC 200 mg/d, then terbinafine 250 mg/d (for 75 d), Changed to POS 800 mg/d for one week/month (continued for 4 months)	Survival	[78]
17	78/F	*F. proliferatum*	None/right total hip arthroplasty replacement	No	FLC (400 mg twice a day at D1 then once a day from D2)	Fungemia	Oral VRC (400 mg twice/d, then (200 mg twice/d) for 72 d	Recovered	[79]
18	38/F	*F. solani*	Kidney transplant, DM	ND	No	Invasive/peritoneal fluid	AmB (50 mg/d)	Alive	[80]
19	65/M	*F. andiyazi*	AML	Yes	Oral POC (3 × 200 mg/d)	Disseminated lung infection	AMB; (3 mg kg/d)	Died	[35]
20	48/F	*F. petroliphilum*	ALL	Yes	No	Fungemia/skin lesions	AMB; (3 mg/kg/day)	Died	[40]
21	ND	*F. temperatum*	None/trauma with maize plant	No	No	Keratitis	Topical natamycin 5% + ITC 200 mg/d	Improved, No follow up	[81]
22	74/M	*F. verticillioides*	Diabetes mellitus	No	No	Fungemia	ND	Died	[82]
23	60/F	*F. napiforme*	Stage III multiple myeloma	Yes	AmB deoxycholate	Fungemia/skin lesions	AMB deoxycholate/for one month	Died	[64]
24	67/M	*F. solani*	Acute biphenotyic pneumonia	Yes		Fungemia/skin lesions/pancytopenia	LAmB (3 mg/kg/day)	Died	[36]
25	21/M	*F. solani*	Multiple organ injury	?	FLC (200 mg/d)	Fungemia	None	Died	[83]
26	65/M	*F. solani*	AML	Yes	ITC	Disseminated/endocarditis/skin lesions	5 mg/kg/d + VRC (4 mg/kg for 25 d, then AmB + Terbinafine (500 mg/d) + GM-CF	No relapse on maintenance therapy	[84]
27	29/M	*F. subglutinans*	None	No	No	Mycetoma, osteomyelitis	ITC 200 mg twice daily for 4 months	Improved	[85]
28	14/M	*F. solani*	Ocular trauma	No	No	keratitis	VRC (10 mg/mL) every hour + topical natamycin (5%) five times daily, + with 500 mg oral ketoconazole twice a day at 12-h intervals (1 g/day).	Improved	[86]
29	52/M	*F. solani*	Corneal injury	No	No	Keratitis and endophthalmitis	Topical 5% natamycin + 0.15% AmB + oral FLC 200 mg/d, And vitrectomy, AmB injection, Then topical 1% VRC + 200 mg twice daily + POS 200 mg four times daily	Infection persist	[87]
30	36/F	*F. prolifertarum*	Lung transplan	No	ITC	Lung infection	L AmB, VRC	Died	[67]
31	30/F	*F. verticillioides*	Liver transplant	?	FLC	Fungemia/skin lesions	VRC 6 mg/kg (360 mg) bid, followed by 4 mg/kg (240 mg) bid for 20 days , then oral(200 mg bid) for a further 5 weeks	alive	[88]
32	27/M	*F. solani*	Cutaneous T cell lymphoma with leukemia	Yes	FLC 400 mg/d (loading dose 800 mg)	Fungemia/skin lesions/Lung infection	AmB deoxycholate + VRC 4.5 mg/kg every 12 h. Discharged on oral VRC + Granulocyte transfusion	Alive after 6 months	[89]

AmB: Amphotericin B; VRC: voriconazole; POS: posaconazole; FLC: fluconazole; AML: acute myeloid leukemia; ALL: acute lymphoblastic leukemia; ?: Data not available.

**Table 2:** Reported MIC values of *Fusarium* species of 9 antifungal agents reported for clinical cases presented in Table 1.

Case No.	*Fusarium* spp.	Fusarium Species Complex	MIC/MEC (µg/mL)									Ref No.
			AmB	FLC	ITC	VRC	POS	ISV	CAS	MCA	ANI	
1	*F. ramigenum*	FFSC	1		>16	2	1	4		>8	>8	[71]
2	*F. petroliphilum*	FSSC	4		>32	4	>32					[72]
3	*F. solani*	FSSC	4	>256	>16	16	>32		>32	>32	>32	[9]
4	*F. keratoplasticum*	FSSC	1	>64	4	4	>16	8		>16	>16	[73]
5	*F. pseudensiforme*	FSSC	0.5	>64	>16	8	4	8		>16	>16	
6	*F. oxysporum*	FOSC	1	>64	>16	8	>16	8		>16	>16	[74]
7	*F. solani*	FSSC	4			8	8					[75]
8	*F. solani*	FSSC	2–4	>64	>8	0.25–0.5	2–8		>16	>16		[76]
9	*F. sacchari*	FFSC	0.5	>64	≥16	≥16			>64		≥16	[77]
10	*F. sacchari*	FFSC	0.5	>64	≥16	4			>64		≥16	
11	*F. sacchari*	FFSC	1	>64	4	0.0625			16		≥16	
12	*F. sacchari*	FFSC		>64	>16	4			>64		≥16	
13	*F. petroliphilum*	FSSC	2	>128	>4	>16	>16		8	4	>16	[29]
14	*F. petroliphilum*	FSSC	1	>128	>4	>16	>16		8	>16	>16	
15	*F. solani sensu lato*	FSSC	0.5	>128	>4	>16	>16		8	4	>16	
16	*F. falciforme*	FSSC	0.5	>64	>16	8	0.5			>8		[78]
17	*F. petroliphilum*	FSSC	6		12	0.75	0.25		>32			[79]
18	*F. solani*	FSSC	2	≥64	≥16					≥8		[80]
19	*F. andiyazi*	FFSC	8	16	8	2	1	4	8	>8		[35]
20	*F. petroliphilum*	FSSC	1	>64	>16	8	>16	>16		>8	>8	[40]
21	*F. temperatum*	FFSC	0.5	>64	>16	1	0.25	4		0.031	4	[81]
22	*F. verticillioides*	FFSC	>32		>32	1	32			>16	>16	[82]
23	*F. napiforme*	FFSC	2–4	1–2	>8	4				>16		[64]
24	*F. solani*	FSSC	8		>8	0.12						[36]
25	*F. solani*	FSSC	1.5	>256	>32	2			>32			[83]
26	*F. solani*	FSSC	1	>4	>8	>8				>16		[84]
27	*F. subglutinans*	FFSC		≥64	≥16		≥16		≥16		≥32	[85]
28	*F. solani*	FSSC	0.5	>64	>16	8	>16		>16		>16	[86]
29	*F. solani*	FSSC	4	>64	>16	>16	8					[87]
30	*F. prolifertarum*	FFSC	4	>128	>128				128			[67]
31	*F. verticillioides*	FFSC	8-16	>256	32	4			≥32			[88]
32	*F. solani*	FSSC	1			4						[89]
Range (total)			0.5–16	16– > 256	4– > 128	0.0625– > 16	0.5– > 32	4– > 16	8– > 128	0.031– > 32	4– > 32	

Abbreviations: FSSC: *F. solani* species complex; FFSC: *F. fujikuroi* species complex; FOSC: *F. oxysporum* species complex. AmB: Amphotericin B; FLC: fluconazole; ITC: itraconazole; VRC: voriconazole; POS: posaconazole; ISV: isavuconazole; CAS: Caspofungin; MCA: megafungin; ANI: anidulafungin.
